# Genome Sequence of the Diploid Yeast Debaryomyces hansenii TMW 3.1188

**DOI:** 10.1128/mra.00649-22

**Published:** 2022-10-26

**Authors:** Tobias Link, Rebekka H. Lülf, Marina Parr, Maik Hilgarth, Matthias A. Ehrmann

**Affiliations:** a Lehrstuhl für Mikrobiologie, Technische Universität München, Freising, Germany; b Department of Bioinformatics, Wissenschaftszentrum Weihenstephan, Technische Universität München, Freising, Germany; Vanderbilt University

## Abstract

Debaryomyces hansenii TMW 3.1188 is a halotolerant diploid yeast that was isolated from lupine moromi fermentation. Here, we report on the 24.77-Mbp genome of a diploid strain of the species D. hansenii.

## ANNOUNCEMENT

**FIG 1:**
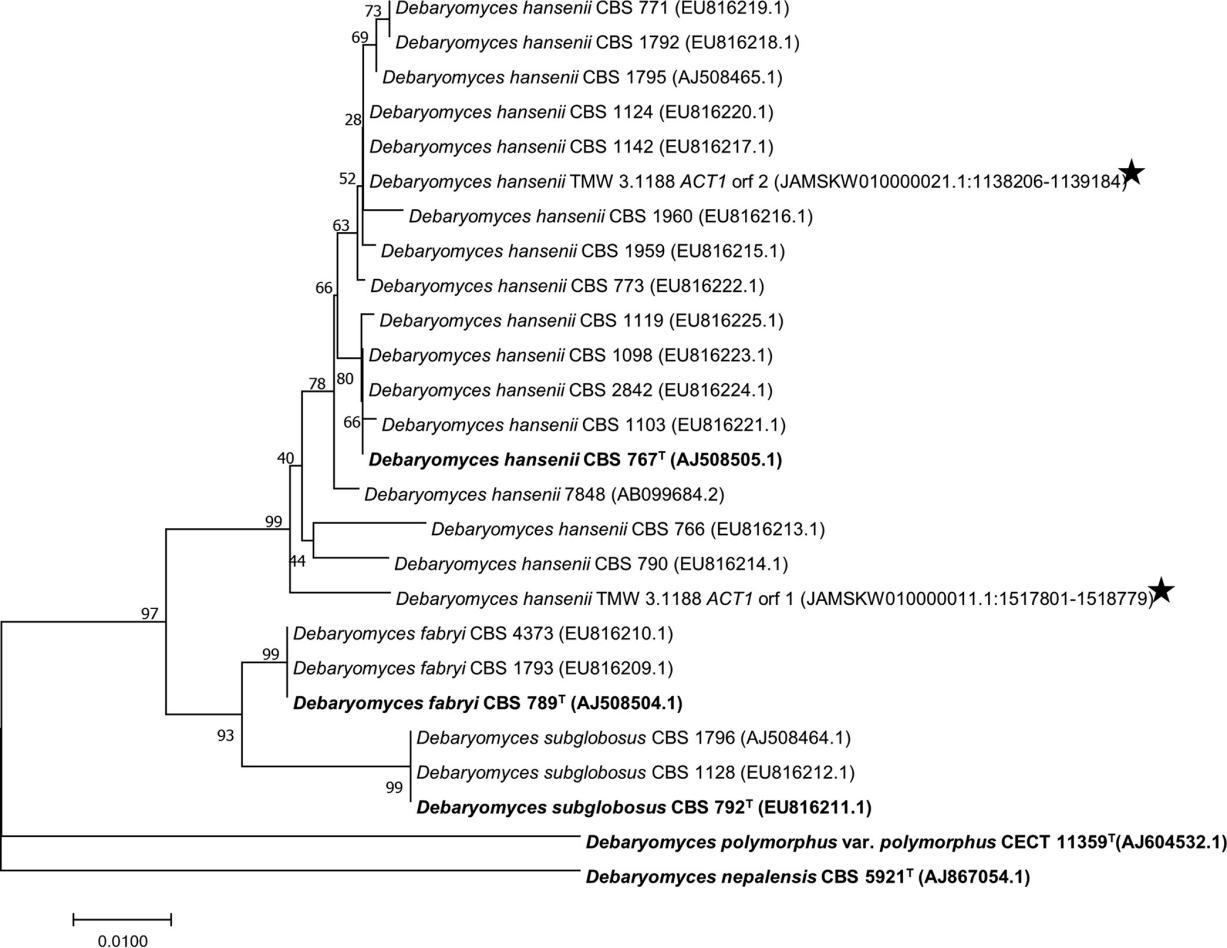
Phylogenetic tree of *Debaryomyces* species. The evolutionary history was inferred using the neighbor-joining method (15). The optimal tree, with the sum of branch lengths of 0.23095609, is shown. The percentages of replicate trees in which the associated taxa clustered together in the bootstrap test (1,000 replicates) are shown next to the branches (16). The tree is drawn to scale, with branch lengths in the same units as the evolutionary distances used to infer the phylogenetic tree. The evolutionary distances were computed using the maximum composite likelihood method (17) and are in the units of the number of base substitutions per site. All positions containing gaps and missing data were eliminated. There were a total of 752 positions in the final data set. Evolutionary analyses were conducted in MEGA7 (18). Type strains were indicated by bold letters with superscript T. Black stars indicate the positions of the sequences from TMW 3.1188. The accession numbers and positions in the chromosome are indicated in parentheses.

Debaryomyces hansenii is a yeast that is commonly found in marine environments or in food fermentations such as cheese or doenjang (1–5). Strain TMW 3.1188 was isolated from a spontaneous lupine moromi fermentation with 10% NaCl (wt/vol) after 12 weeks (6). The identity of the isolate was initially verified via 28S rDNA sequencing using the primers V9G (7) and LR5 (8) (GenBank accession number OP179623). However, because the *ACT1* gene was reported to have greater variability within *Debaryomyces* species, both alleles from TMW 3.1188 were used to verify the affiliation with the D. hansenii species (Fig. 1) (9, 10). Isolation of the genomic DNA of TMW 3.1188 was carried out with harvested cells that had been grown for 24 h at 30°C in DSMZ 90 medium with 5% NaCl (wt/vol). DNA was isolated using R-zymolyase (Zymo Research), followed by phenol-chloroform extraction and enzymatic digestion of proteins with proteinase K.

Quantification and fragment distribution analysis of the DNA were performed with a Qubit fluorometer (Life Technologies) and a Femto Pulse electrophoresis system (Agilent). The library was created with the SMRTbell Express template preparation kit v2.0 (Pacific Biosciences [PacBio]). The library was size selected to >17.5 kbp using a BluePippin device (Sage Science) and then sequenced using the Sequel sequencing kit v3.0 (PacBio) according to the manufacturer’s instructions. The read mode was set to continuous long reads (CLR) on a PacBio Sequel instrument.

A total of 10,724,419,855 bases were sequenced. The mean subread length was 14,003 bases, with an *N*_50_ value of 19,076 bp. The mean of the longest subreads was 14,126 bases, with an *N*_50_ value of 19,076 bp. After sequencing, data quality was checked via the PacBio single-molecule real-time (SMRT) Link software. The genome was assembled with HGAP4 in SMRT Link v10.0.0.108728 with default parameters, except that the estimated genome size was set to 12 Mbp. The assembly generated 26 contigs, with a total sequence length of 24,773,645 bp, an *N*_50_ value of 1,604,673 bp, and a GC content of 36.23%.

Genomic comparisons using FastANI v1.3 (11) and genomediff in GenomeTools v1.6.2 (12) revealed that TMW 3.1188 has the greatest similarity to strain CBS767^T^ (GenBank assembly accession number GCF_000006445.2), with an average nucleotide identity (ANI) value of 95.72%, while the *K*_r_ distance, which estimates the number of substitutions per site between two unaligned DNA sequences, has a value of 0.055 (13). The ANI value with respect to the type strain of the species Debaryomyces fabryi, CBS789^T^ (GenBank assembly accession number GCF_001447935.2), is 85.33%, and the *K*_r_ distance is 0.102. The ANI values with respect to strains J6 (GenBank assembly accession number GCA_001682995.1) and MTCC 234 (GenBank assembly accession number GCA_000239015.2) are only 84.5%. The *K*_r_ distance from TMW 3.1188 to J6 is 0.119 and that to MTCC234 is 0.129.

Furthermore, genomic comparison using the D-GENIES (14) online tool revealed that each chromosome of CBS767^T^ had two contigs of TMW 3.1188 aligning to it, one with a higher ANI value (>57%) and one with a lower ANI value (34 to 40%), proving the existence of two chromosomal sets. Contigs 10, 12, 13, and 26 of TMW 3.1188 align to the mitochondrial genome of Debaryomyces hansenii (GenBank assembly accession number GCF_000006445.2).

### Data availability.

The assembled genome is available under assembly accession number GCA_024256405.1, with BioProject accession number PRJNA841823. The raw reads are available under SRA accession number SRR19753073. The 28S rDNA sequence is available under GenBank accession number OP179623.
